# Evaluation of the effects of sea tangle water extract and various packaging methods on the product quality of pre- and post-rigor cured pork loin during storage

**DOI:** 10.5713/ab.25.0061

**Published:** 2025-06-24

**Authors:** Haeun Kim, Koo Bok Chin

**Affiliations:** 1 Department of Animal Science, Chonnam National University, Gwangju, Korea

**Keywords:** Modified Atmosphere Packaging, Post-rigor, Pre-rigor, Sea Tangle, Vacuum Packaging

## Abstract

**Objective:**

This experiment evaluated the physicochemical properties of low-salt cured pork after adding hot-water-extracted sea tangle (HWEST; 5%, 10%) to pre-rigor loin and packaging with vacuum packaging (VAC) or modified atmosphere packaging (MAP; 70% CO_2_, 20% N_2_) over an 8-wk storage period at 10°C.

**Methods:**

Pre-rigor cured loin (<1 h post-slaughter) treated with 0.8% salt and 5%/10% HWEST was prepared and compared to post-rigor cured loin with a salt level of 1.5%. The pH, color values, Warner–Bratzler shear value (kgf), lipid oxidation (thiobarbituric acid reactive substances), volatile basic nitrogen, cooking and purge loss (CL, PL %), expressible moisture (EM, %), and microbial counts of the cooked loin were measured.

**Results:**

MAP of cured pork loin yielded a lower PL value than VAC. In addition, lactic acid bacteria did not grow in MAP during storage. The 0.8%-salt pre-rigor cured loin treated with HWEST (5%, 10%) exhibited the same CL as the 1.5%-salt post-rigor cured loin. VAC elicited no differences in physicochemical properties between the 1.5%-salt post-rigor cured loin and HWEST-treated 0.8%-salt pre-rigor cured loin after the 8-wk storage period. However, the 0.8%-salt pre-rigor cured loin treated with HWEST (5%, 10%) yielded the same EM value as the 1.5%-salt post-rigor cured loin after 8 wk of VAC storage.

**Conclusion:**

The low salt content of meat products can be compensated for using pre-rigor cured loin, HWEST, and MAP.

## INTRODUCTION

The salt in processed meat products affects consumer palatability, and the interaction between protein and fat improves meat product texture and yield by mitigating moisture loss during cooking. It is known that the lower the salt content, the weaker the flavor, since salt affects the enhancement of sweet and volatile flavor compounds and suppression of bitter taste [1]. Therefore, flavor enhancement and functionality should be considered when developing low-salt meat products. Salt also reduces the water activity of meat products and inhibits microbial growth. Accordingly, low-salt foods are more vulnerable to damage during storage; thus, product shelf-life extension should be considered in this regard.

Various packaging methods for storing meat products are available. In the modified atmosphere packaging (MAP) method, the air inside the packaging is controlled by gas mixtures, comprising gases such as nitrogen, carbon dioxide, and oxygen, in product-specific ratios. This packaging method can delay biochemical spoilage. However, vacuum packaging (VAC) completely removes the air inside the packaging, thus decelerating fat and protein oxidation owing to the absence of oxygen. It is characterized by a sour odor, and the lack of oxygen can cause the myoglobin in meat to become deoxymyoglobin, which turns the meat dark red, thus negatively affecting consumer acceptance [2]. However, MAP can circumvent the VAC-induced dark-red coloration of meat and minimize the effect of fat and protein oxidation delay. Moreover, it potentially addresses the negative aspects of aerobic and VAC. Cases of MAP application to meat products have been reported [3–6].

Sea tangle contains glutamic acid, Na, and K, which are known to enhance umami flavor [7]. These components make it suitable for improving flavor and functionality in low-salt meat products. Moroney et al [8] reported that seaweed extract improved the storage stability of pork patties without affecting color. Kim et al [9] also found that sea tangle powder enhanced the texture of low-salt sausages. Therefore, based on these characteristics, hot-water-extracted sea tangle (HWEST) was selected and applied in this study as a natural additive to compensate for reduced salt content and to improve water-holding capacity in cured pork loin.

Pre-rigor muscle has a higher temperature and pH than its post-rigor counterpart [10]. Meat product manufacture using pre-rigor muscle has been found to improve salt-soluble protein extraction, cooking loss (CL), and water-holding capacity [11]. Owing to pre-rigor muscle’s characteristics, its utilization in low-salt meat products reportedly conveys numerous advantages [12].

Although previous studies have reported the individual effects of seaweed extracts or packaging methods, such as MAP, on meat product quality, studies that concurrently apply low-salt formulation (0.8% NaCl), HWEST, and pre-rigor pork in combination with different packaging systems remain limited. The present study applied this multifactorial approach and demonstrated that HWEST-treated pre-rigor pork loin cured with reduced salt could achieve comparable physicochemical and water-holding properties to conventional post-rigor products. Furthermore, MAP appeared to be effective in reducing purge loss (PL) and suppressing LAB growth during storage.

## MATERIALS AND METHODS

### Preparation of sea tangle extract and pork loin

The sea tangle used for the brine solution was purchased from a local food market. To prepare HWEST, 2,700 mL of double-distilled (dd) water was added to 300 g of sea tangle, and the resulting mixture was boiled, extracted for approximately 2 h, filtered through a 500-μm sieve (ChungGye Industrial MFG.) and stored at 4°C for use as brine solution for pork loin. The pork loin was procured from a local meat market (Hyeondaeyootong). Pre-rigor pork loin was obtained within 1 h after slaughter, whereas post-rigor pork loin was used 1 day after slaughter. After removing excess fat and connective tissue, pre- and post-rigor pork loin was sectioned into 6-cm-thick slices and subjected to curing.

### The manufacture and packaging of pork loin

Brine solution was injected to achieve an 0.8% salt concentration in raw pre-rigor pork loin, and 0%, 5%, and 10% HWEST was subsequently injected into each pork loin sample. Post-rigor pork loin was prepared by injecting 1.5%-salt brine solution. The formulation of the brine solution is illustrated in Table 1. The brine solution was injected into raw meat via multi-needle injection (Seasoning Injector; Geebake) after 30 min to allow effective permeation of the brine solution. Thereafter, the pork loin was cooked in a 75°C water bath until its internal temperature reached 70°C. After cooking, the pork loin was stored on ice. The cooked, HWEST-treated pork loin was further divided into two packaging-based groups: VAC and MAP (70% CO_2_, 20% N_2_). A polypropylene+linear low-density polyethylene (263×200–32 mm; HyperCac) packaging container was used, and 25-μm thick polyethylene film (Sealed Air File; HyperVac) was applied. Nylon+polyethylene terephthalate (15 μm, 45 μm, 400×650) vacuum sealer bags were used for VAC. The pork loin was refrigerated at 10°C, and alterations in its physicochemical properties according to storage period (0, 2, 4, and 8 wk) were measured. The 10°C storage was used as an accelerated condition to induce spoilage and monitor changes in physicochemical and microbial properties over time [13]. Although this temperature does not represent typical commercial cold storage, it was selected to evaluate the effects of packaging under stress conditions.

### pH and color values

The pH values of pre-and post-rigor raw or cooked pork loin were repeatedly measured five times at various parts using a Minolta pH meter (MP-120; Mettler-Toledo), and the average of the five measurements made at the different locations was obtained. Meat surface color was measured six times and expressed as an average value. Lightness (L*), redness (a*), and yellowness (b*) were measured using a colorimeter (CR-10; Minolta). The CIELAB color values for a standard white plate were L* = 92.4, a* = 1.9, and b* = 0.7.

### Purge loss

PL was determined by measuring weight before and after storage of the 10°C refrigerated samples. PL values were calculated by applying the following formula:


(1) $$\text{PL }(\%)=\frac{\left(\text{Weight of the pork loin before storing}-\text{Weight of the pork loin after storing}\right)}{\text{Weight of the pork loin before storing}}\times 100$$

### Cooking loss (%) and expressible moisture

CL (%) was evaluated by measuring sample weight before cooking and subsequently removing moisture from the sample’s surface after cooking. Sample CL values were expressed as a percentage (%) using the following formula:


(2) $$\text{CL }(\%)=\frac{\left(\text{Weight of the pork loin before cooking}-\text{Weight of the pork loin after cooking}\right)}{\text{Weight of the pork loin before cooking}}\times 100$$

Expressible moisture (EM; %) was measured via centrifugation, as described by Jauregui et al [14]. Three pieces of filter paper (Whatman #3, #Cat No 1003-110) were divided into quarters and weighed. Cooked pork loin samples were cut into 1.5-g cubes and wrapped in filter paper (Whatman No. 3 #Cat No. 1003-110). After sample centrifugation at 1,660×g for 15 min, each sample’s EM value was expressed as a percentage (%) and calculated as the weight difference before and after centrifugation.

### Thiobarbituric acid reactive substances

Lipid oxidation in pre- and post-rigor pork loin was evaluated using a UV–visible spectrophotometer (X-Ma 1200) at 532 nm, and the absorbance of thiobarbituric acid reactive substances (TBARS) was measured according to Sinnhuber and Yu [15]. TBARS values were expressed in mg malondialdehyde (MDA)/kg of meat sample.

### Volatile basic nitrogen

Volatile basic nitrogen (VBN) was measured using the method of Conway [16], with slight modifications. Pork loin samples (1 g) were each homogenized for 30 s with 9 mL of dd water. Each homogenized sample was filtered through filter paper (Whatman No. 2 #Cat No. 1002-110). After 1 mL of filtrated sample had been transferred to the outer Conway chamber, 1 mL of 0.01 N boric acid solution and 50 μL of indicator (bromocresol green, 0.066%+methyl red, 0.066%) were added to the inner Conway chamber. In the outer Conway chamber, 1 mL of K_2_CO_3_ solution (50%) was added and stirred horizontally. After incubation at 37°C for 2 h, 0.01 N HCl was titrated into the inner Conway chamber, and data were expressed in mg%.

### Warner–Bratzler shear value

The shear value represents the average of 10 measurements of the cooked samples with the following dimensions: 1×1×4 cm in the muscle fiber direction. It was measured using an Instron Universal Testing Machine (Model 3344) and expressed in units of force required to cut the sample. The shear blade speed was set to 300 mm/min and 500 N.

### Microbial counts

Microbial analysis was performed on the pre-and post-rigor pork loin samples stored for 0, 2, 4, and 8 wk at 10°C. Each sample (10 g) was mixed with 90 mL of sterilized dd water and homogenized using a lab blender (BagMixer 400; Interscience) for 20 s. Microbial counts were conducted using total plate count (TPC) agar (Difco, Detroit, Mich), violet red bile (VRB) agar (Difco) for Enterobacteriaceae, and de Man–Rogosa–Sharpe agar (Difco) for LAB. These plate agars were incubated at 37°C for 48 h. After incubation, all the colonies were expressed as the logarithm of colony-forming units (log CFU/g sample).

### Statistical analysis

All experiments were conducted in triplicate, and the results are expressed as the mean±standard deviation. Using SPSS 22.0 (version 25; IBM), one-way analysis of variance (ANOVA) of pH and color values before and after cooking was performed on each treatment. After the pork loin had been cooked, two-way ANOVA was performed according to storage period (0, 2, 4, and 8 wk) and treatment as factors. Duncan’s multiple-range test was conducted to test the significance of differences among means at the 5% significance level (p<0.05).

## RESULTS AND DISCUSSION

### pH values and temperature of raw pre- and post-rigor pork loin muscle

The pH values and temperature of pre- and post-rigor pork loin are shown in Table 2. Apparent pH and temperature differences between pre- and post-rigor meat were observed in this study. As shown in Table 2, the pH values and temperature of pre-rigor pork loin exceeded those of its post-rigor counterpart (p<0.05). Pre-rigor pork loin had a higher pH, which exceeded the isoelectric point, and more space existed among the muscle fibers to hold more moisture, resulting in improved functional properties, such as water-holding capacity, salt-soluble protein extraction, and processing yield [10]. The absorption capacity of the brine solution, processing yield, and protein solubility were higher in pre-rigor muscle than in post-rigor muscle [17].

### Quality characteristics of hot-water-extracted sea tangle-injected cured pork loin

*pH and color values:* Table 3 shows the pH and color values of HWEST-injected raw pork loin. Post-rigor pork loin exhibited lower pH values than pre-rigor pork loin (p<0.05), displaying a similar scenario to that in raw meat before injecting the brine solution. Ham et al [17] reported that the pH values of pre-rigor poultry meat cured for 7 days exceeded those of its post-rigor counterpart (p<0.05) owing to a higher ionic strength inhibiting glycolytic enzyme denaturation. Claus and Sørheim [10] reported that the pH values of post-rigor beef patties were lower than those of 1.7%-salt pre-rigor beef patties, indicating that this level of salt concentration was insufficient to inhibit glycolysis.

The color values (L*, a*, and b*) of cured pork loin exhibited no differences across all treatments (p>0.05). Therefore, no differences in the state of post- and pre-rigor meat were observed (p>0.05). Keenan et al [18] reported no post-curing differences in the color values of post- and pre-rigor pork loin, as this study confirmed. No differences in color values occurred according HWEST addition (p>0.05). Moroney et al [8] reported that the addition of seaweed extract to pork patties did not affect color values, displaying consistency with this study. However, Kim et al [9] reported that adding sea tangle powder to pork sausage decreased lightness and redness but increased yellowness. They inferred that sea tangle powder affected sausage color. Processing conditions have been found to discolor the total phenols and tannins contained in seaweed, and sea tangle has been found to contain several phenolic and tannin components [19]. Therefore, sea tangle color may not affect cured pork meat products owing to differences in sea tangle-processing procedures.

### Quality characteristics of hot-water-extracted sea tangle-injected pork loin after cooking

*pH and color:* Table 4 shows the pH and color values of HWEST-injected cooked pre- and post-rigor pork loin. No differences in pH were noted according to treatment and packaging method (p>0.05). Depending on storage period, 4-wk storage yielded the highest pH values. However, with 8-wk storage, pH tended to decrease (p<0.05). Various factors have been found to decrease the pH value of meat. Among these factors, lactic acid was observed to reduce pH [20]. Evidently, the LAB count in this study peaked at 8 wk of storage (Table 5); hence, the decreased pH values at 8 wk were considered to be affected by LAB.

Color measurements revealed no difference in lightness according to treatment (p>0.05). Pre-rigor treatments exhibited higher redness and yellowness values than post-rigor treatments (p<0.05). Pre-rigor pork loin samples displayed higher redness and yellowness values than their post-rigor counterparts because of pre-rigor muscle’s lower metmyoglobin content than that of post-rigor muscle [21]. Pre-rigor pork loin had higher oxymyoglobin levels than post-rigor pork loin, explaining why pre-rigor meat exhibited a higher redness value than post-rigor meat [22]. In addition, color may depend on differences in the salt concentration applied to the pork loin. The higher the salt content, the greater the extraction of sarcoplasmic proteins from the meat’s surface, thereby increasing redness [23]. Fernández-López et al [24] reported that redness increased with increasing salt concentration because salt increases the concentration of myoglobin by diminishing the moisture on the surface of the meat. However, in this study, low-salt-concentration treatments yielded higher redness values; therefore, the effect of the carcass itself was more notable than that of the salt concentration. No difference was noted according to the quantity of HWEST added (p>0.05), exhibiting consistency with a previous study [7] wherein adding HWEST to cooked ham products did not affect their color. In this study, no differences in lightness, redness, and yellowness were observed according to storage period (p>0.05).

*Purge loss (%):* As shown in Table 4, MAP exhibited less PL than VAC (p<0.05). Since no difference was noted according to treatment within the same packaging method (p>0.05), pork loin PL was considered unaffected by carcass state (pre vs. post), brine solution salt concentration, and HWEST. Kameník et al [5] reported that VAC increased PL from storage day 7 in pork meat stored under MAP and VAC conditions for 35 days, displaying consistency with this study’s results. MAP potentially elicits different storage characteristics depending on the ratio of gases injected during packaging. An air composition comprising 100% CO_2_ may attenuate the water-holding capacity of pork loin [25]. Addition of CO to the CO_2_ and N_2_ gas mixture may alter meat product quality during storage [26]. In this study, using MAP (CO_2_:N_2_ = 30:70) potentially mitigated PL-related product yield loss during storage and inhibited microbial growth.

*Thiobarbituric acid reactive substances (mg of malondialdehyde/kg):* Table 4 shows the TBARS results of pork loin. The TBARS value ranged from 0.32 to 0.34 MDA/kg in all treatment groups and did not differ among treatments (p>0.05). This study was congruent with that of Torres et al [22]. Zhang and Sundar [27] found that when packaging fresh pork meat, the packaging method involving an oxygen content>25%–55% yielded a TBARS value≥0.5 mg MDA/kg meat, while that involving 5%–25% oxygen exhibited no difference in TBARS value across samples. In this study, determining differences among treatments was considered difficult because all treatments were stored in low-oxygen packaging (MAP or VAC). In addition, Juncker et al. [12] reported that when the pH value of meat was <5.8, lipid oxidation was high; however, a pH value>5.8 did not significantly affect the TBARS value. Since the pH of the cooked treatments in this study ranged from 5.93 to 6.04, no differences in TBARS value presumably occurred across treatments. According to storage period, high TBARS values were generated at 8 wk of storage (p<0.05) compared with those at other storage periods (0, 2, and 4 wk). A TBARS value≥0.6 mg MDA/kg reportedly elicited a general off-flavor perception among consumers [28]. However, in this study, a TBARS value of 0.5 mg MDA/kg was generated at 8 wk of storage. Lipid oxidation-induced quality deterioration in pork loin was confirmed to be minimal in this study. Although HWEST contains alginate and other water-soluble components with antioxidant activity [19], no effect on lipid oxidation was observed under the current conditions. The consistently low TBARS values across all treatments are likely due to the low-oxygen packaging environment (MAP and VAC), which likely limited lipid oxidation, irrespective of HWEST treatment.

*Volatile basic nitrogen (mg%):* VBN can be used to determine the effect of experimental treatments on proteins. As shown in Table 4, no difference in VBN content was noted among all the treatments (p>0.05). Packaging method, pork carcass state, and HWEST addition did not affect the VBN results. Zhang and Sundar [27] found that the lower the oxygen concentration when packaging pork loin, the higher the VBN content under the influence of Pseudomonas; moreover, the higher the CO_2_ content, the lower the microbial load, such as that of Pseudomonas, thus resulting in lower VBN content. No difference in VBN content presumably occurred among the treatments according to packaging method. According to storage period, higher VBN values were observed at 4 and 8 wk of storage than at 0 wk (p>0.05), albeit lower than the VBN threshold level of meat products (20 mg%). The constituent protein was degraded by enzymatic and bacterial activity, influencing high VBN values [29]. This study’s microbial results revealed that the TPC and LAB values increased with the storage period (Table 5), a phenomenon considered to have influenced the VBN results.

*Warner–Bratzler shear value (kgf):* Table 4 shows the Warner–Bratzler shear values (WBSVs) of cooked post- and pre-rigor pork loin treated with HWEST. The WBSV did not differ across all treatments (p>0.05). In this study, HWEST, differences according to carcass state (post- and pre-rigor), and salt concentration did not appear to affect product tenderness. A difference in tenderness may exist according to muscle condition (pre- or post-rigor). However, no difference in shear force was noted depending on the brine solution injected into the carcass [18]. Duranton et al [30] reported that in 3%-salt pork ham, the brine solution enhanced tenderness owing to the protein hydration effect, which was increased by ionic action. The salt concentration of the brine solution used in this study was <1.5%; thus, no difference presumably occurred according to salt concentration. Barbieri et al [7] reported that adding seaweed extract to 1.0%- and 1.2%-salt brine solution in cooked pork ham did not affect the WBSV, displaying consistency with this study’s results.

**Figure 1:**
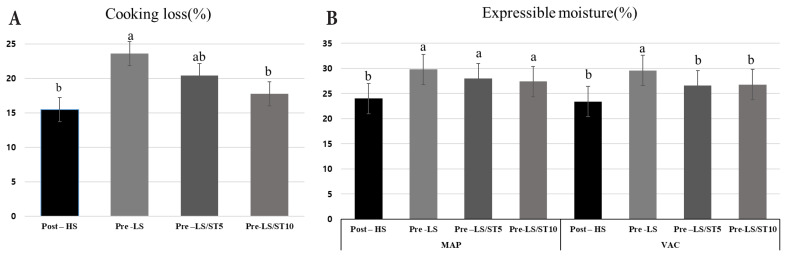
Cooking loss (CL, %) (A) and expressible moisture (EM, %) (B) of pre- and post-rigor cooked pork loins. ^a,b^ Different letters indicate significant differences among treatments (p<0.05). Treatment: Post-HS = post-rigor-manufactured pork loin ham containing 1.5% salt; Pre-LS = pre-rigor-manufactured pork loin ham containing 0.8% salt; Pre-LS/ST5 = pre-rigor-manufactured pork loin ham containing 0.8% salt and 5% sea tangle extract; Pre-LS/ST10 = pre-rigor-manufactured pork loin ham containing 0.8% salt and 10% sea tangle extract. MAP, modified atmosphere packaging; VAC, vacuum packaging.

*Cooking loss (%) and expressible moisture (%):* Figure 1 shows the CL and EM results of HWEST-treated pre-rigor and 1.5%-salt post-rigor pork loin. 0.8%-salt pre-rigor pork loin treated with 5%, and 10% HWEST exhibited no differences in CL from 1.5%-salt post-rigor pork loin (p>0.05). In MAP, HWEST treatment (Pre-LS/ST5, Pre-LS/ST10) elicited higher EM values than Post-HS (p<0.05). 1.5%-salt post-rigor pork loin, 0.8%-salt pre-rigor pork loin treated with 5% and 10% HWEST yielded similar CL values within the VAC method (p>0.05). Even 5% HWEST addition to 0.8%-salt pre-rigor pork loin ostensibly improved EM and alleviated CL. In general, the higher the salt concentration, the higher the salt-induced solubility of myofibrillar protein, thereby improving the fat and water retention capacity. Consequently, organizational characteristics improve, leading to increased water-holding capacity [31]. However, in this study, pre-rigor pork loin injected with 0.8% salt and HWEST displayed a water-holding capacity similar to or higher than 1.5% post-rigor pork loin. The reason for this result can be interpreted in two ways. First, the pre-rigor approach is assumed to increase functionality. In this study, cooked pre-rigor pork loin yielded higher pH values than cooked post-rigor pork loin (p<0.05). When the pH values of the meat approximated the isoelectric point (PI) of 5.5, the number of positive and negative charges equalized, weakening the water-holding capacity. However, pH values distant from the PI are associated with increased space to retain water [32]. As the pre-rigor pork loin used in this study had a pH value farther from the PI than its post-rigor counterpart, thus having more space to retain water, its water-holding capacity was considerably elevated, even at a low salt concentration. The extracellular space surrounding muscle fibers increased up to 24 h after slaughter, and the spacing between muscle fiber bundles decreased between 9 and 24 h post-slaughter [33]. Accordingly, the use of pre-rigorous raw meat is considered to affect the water-holding capacity of low-salt pork loin. Second, the improvement in water retention is likely due to the presence of alginate in HWEST. Alginate, a major soluble component in sea tangle, forms a gel matrix in the presence of calcium ions [34], stabilizing water within the muscle through electrostatic and hydrogen bonding interactions [35]. These interactions generate a protein–polysaccharide network [36], which reduces moisture loss during heating and storage. In this study, Pre-LS using pre-rigor loin yielded a higher CL and lower EM value than other treatments (p<0.05). Rather than the independent effect of the carcass’s state, combined pre-rigor and HWEST treatment simultaneously yielded a distinct water-holding capacity. In addition, the MAP condition itself may contribute to the reduced PL observed in HWEST-treated pork loin. CO_2_ in the gas mixture can stabilize muscle proteins and reduce denaturation during storage, thereby improving water-holding capacity and minimizing moisture loss [25,31].

*Microbial counts:* The total counts of bacteria, Enterobacteriaceae, and LAB according to pre- and post-rigor pork loin storage period were enumerated (Table 5). The TPC and VRB results did not differ among treatments (p>0.05). TPC increased with storage time (p<0.05), while VRB displayed no difference <2 CFU/g on all storage days (p>0.05). Although differences emerged in the number of LAB according to packaging method (p<0.05), variations in pre- and post-rigor differences, sea tangle extract addition, and salt concentration did not affect the LAB count (p>0.05). Moreover, VAC elicited a higher LAB count than MAP (p<0.05). Metaxopoulos et al [4] reported that meat products subjected to MAP with an air ratio of 80% CO_2_ and 20% N_2_ exhibited less LAB growth than VAC meat products, displaying a trend similar to that in this study. LAB cause product spoilage under oxygen-free conditions and refrigerated storage, culminating in product quality deterioration, such as a sour odor, gas generation, and slime formation [19]. This inhibitory effect can be attributed to the presence of CO_2_ in the MAP environment, which penetrates bacterial cells, lowers intracellular pH, and disrupts enzymatic activity required for microbial metabolism [4,20]. These changes suppress LAB growth, thereby extending shelf-life. The TPCs remained below the microbial specification limit of 7 log CFU/g for fresh meat [37], indicating microbiological safety and shelf-life stability of the samples. Additionally, suppression of LAB activity under MAP conditions may help prevent quality deterioration caused by ropy slime and other spoilage traits commonly observed in vacuum-packed meat products [20].

## CONCLUSION

This study evaluated the physicochemical properties of 1.5%-salt post-rigor pork loin compared with those of 0.8%-salt pork loin treated with HWEST (5%, 10%) prepared using pre-rigor pork as a raw material. In addition, it endeavored to develop a pork loin product with improved storage properties and a reduced sodium content through VAC and MAP over an 8-wk storage period at 10°C. Before cooking, cured pre-rigor pork loin yielded higher pH values than cured post-rigor pork loin. After cooking, pre-rigor pork loin exhibited higher a* and b* values than post-rigor pork loin, and HWEST addition did not affect cooked pork loin color. Pre-rigor pork loin treated with HWEST (5%, 10%) displayed the same CL as post-rigor pork loin. MAP elicited lower PL and LAB count values than VAC, depending on the packaging method. In conclusion, the functional and quality-deterioration characteristics of meat products that potentially occur owing to salt reduction can be compensated for using pre-rigor and sea tangle extract treatment. Furthermore, storage can be improved via MAP. These findings indicate that combining pre-rigor raw meat, HWEST, and MAP could aid in developing reduced-sodium pork loin with enhanced storage stability. These strategies may also enable clean-label claims like “reduced salt” or “natural extract added,” satisfying consumer demand for healthier, minimally processed meat products.

## Figures and Tables

**Table 1:** Pre- and post-rigor formulation of pork loin ham brine solution

Ingredients (%)	Treatment^1)^			

	Post-HS	Pre-LS	Pre-LS/ST5	Pre-LS/ST10
Meat	83.30	83.30	83.30	83.30
Ice water	12.73	13.43	8.43	3.43
Salt	1.28	0.58	0.58	0.58
Cured blend^2)^	0.24	0.24	0.24	0.24
Phosphate	0.4	0.4	0.4	0.4
Sodium erythorbate	0.05	0.05	0.05	0.05
Sugar	1.0	1.0	1.0	1.0
Corn syrup solid	1.0	1.0	1.0	1.0
Sea tangle water extract			5.0	10.0
Total	100	100	100	100

1) Treatment: Post-HS = post-rigor-manufactured pork loin ham containing 1.5% salt; Pre-LS = pre-rigor-manufactured pork loin ham containing 0.8% salt; Pre-LS/ST5 = pre-rigor-manufactured pork loin ham containing 0.8% salt and 5% sea tangle extract; Pre-LS/ST10 = pre-rigor-manufactured pork loin ham containing 0.8% salt and 10% sea tangle extract.

2) Cured blend: comprises 93.75% salt and 6.25% sodium nitrite.

**Table 2:** Raw pork loin pH and temperature

	Post-rigor	Pre-rigor
pH	5.72±0.12^b^	6.18±0.04^a^
Temperature	10.2±0.35^b^	24.1±0.13^a^

a,b Mean values with different superscripts in the same row indicate significant differences (p<0.05).

**Table 3:** Pre- and post-rigor raw pork loin ham pH and color values

	Treatments^1)^			

	Post-HS	Pre-LS	Pre-LS/ST5	Pre-LS/ST10
pH value	5.74±0.06^b^	6.00±0.12^a^	6.03±0.09^a^	6.08±0.08^a^
L*	46.3±1.40^a^	45.1±1.11^a^	45.8±0.85^a^	45.3±1.15^a^
a*	2.13±0.85^a^	1.49±0.25^a^	1.59±0.52^a^	1.69±0.86^a^
b*	4.48±0.78^a^	4.57±1.02^a^	4.69±0.79^a^	5.29±0.48^a^

1) Treatment: Post-HS = post-rigor-manufactured pork loin ham containing 1.5% salt; Pre-LS = pre-rigor-manufactured pork loin ham containing 0.8% salt; Pre-LS/ST5 = pre-rigor-manufactured pork loin ham containing 0.8% salt and 5% sea tangle extract; Pre-LS/ST10 = pre-rigor-manufactured pork loin ham containing 0.8% salt and 10% sea tangle extract.

a,b Mean values with different superscripts within the same row indicate significant differences (p<0.05).

L*, lightness; a*, redness; b*, yellowness.

**Table 4:** Pre- and post-rigor cooked pork loin pH, color, PL, TBARS, VBN, and shear force values

Treatment^1)^		pH	L*	a*	b*	PL	TBARS	VBN	WBSV
MAP	Post-HS	6.02±0.14^a^	68.3±1.97^a^	6.00±1.44^c^	4.83±1.91^b^	1.23±0.74^b^	0.34±0.16^a^	10.8±6.98^a^	1.47±0.49^a^
	Pre-LS	6.02±0.19^a^	69.6±3.70^a^	7.86±1.13^ab^	6.67±1.49^a^	1.84±1.16^b^	0.34±0.15^a^	9.46±6.23^a^	2.57±0.65^a^
	Pre-LS/ST5	6.02±0.17^a^	67.6±3.22^a^	8.04±1.22^a^	6.68±1.29^a^	1.79±1.84^b^	0.34±0.16^a^	9.19±5.76^a^	2.17±0.61^a^
	Pre-LS/ST10	6.04±0.14^a^	66.6±2.75^a^	7.79±0.95^ab^	6.88±1.52^a^	1.90±2.15^b^	0.33±0.16^a^	9.82±6.07^a^	2.03±0.69^a^
VAC	Post-HS	5.98±0.22^a^	67.9±2.04^a^	6.68±1.75^bc^	4.66±1.30^ba^	5.06±1.05^a^	0.33±0.14^a^	11.4±8.32^a^	1.68±0.64^a^
	Pre-LS	5.96±0.27^a^	67.4±2.34^a^	8.65±0.66^a^	6.70±1.67^a^	5.52±2.83^a^	0.32±0.15^a^	11.1±7.33^a^	2.44±0.63^a^
	Pre-LS/ST5	5.93±0.26^a^	67.4±3.81^a^	8.00±1.28^a^	6.78±1.68^a^	5.34±2.36^a^	0.32±0.15^a^	9.99±6.27^a^	2.12±0.51^a^
	Pre-LS/ST10	5.97±0.21^a^	66.4±1.98^a^	7.69±0.80^ab^	6.85±1.43^a^	6.20±2.56^a^	0.33±0.16^a^	10.2±6.06^a^	2.10±0.53^a^
Storage day (wk)									
0		5.95±0.09^ab^	67.4±2.12^a^	7.75±1.28^a^	6.89±1.52^a^	Na	0.27±0.10^b^	6.04±1.82^b^	1.72±0.57^a^
2		6.08±0.10^a^	66.4±2.81^a^	7.48±1.24^a^	5.77±2.00^a^	2.44±1.27^b^	0.33±0.12^b^	9.08±6.25^ab^	2.30±0.59^a^
4		6.05±0.03^a^	68.1±3.24^a^	7.65±1.51^a^	5.78±1.39^a^	3.21±1.83^a^	0.31±0.07^b^	12.9±7.61^a^	2.18±0.71^a^
8		5.89±0.35^b^	68.7±2.90^a^	7.47±1.61^a^	6.57±1.77^a^	3.61±2.58^a^	0.43±0.22^a^	13.0±6.11^a^	2.09±0.68^a^

1) Treatment: Post-HS = post-rigor-manufactured pork loin ham containing 1.5% salt; Pre-LS = pre-rigor-manufactured pork loin ham containing 0.8% salt; Pre-LS/ST5 = pre-rigor-manufactured pork loin ham containing 0.8% salt and 5% sea tangle extract; Pre-LS/ST10 = pre-rigor-manufactured pork loin ham containing 0.8% salt and 10% sea tangle extract.

a–c Mean values with different superscripts within the same column indicate significant differences (p<0.05).

TBARS, thiobarbituric acid reactive substances; VBN, volatile basic nitrogen; PL, purge loss; WBSV, Warner–Bratzler shear value; MAP, modified atmosphere packaging; VAC, vacuum packaging; Na, not analyzed.

**Table 5:** Microbial counts ([log cfu/g]) in pre- and post-rigor cooked pork loin

Treatment^1)^		Parameters		

		TPC	VRB	LAB
MAP	Post-HS	2.11±1.04^a^	<2^a^	<2±0.54^c^
	Pre-LS	2.07±0.93^a^	<2^a^	<2±0.72^bc^
	Pre-LS/ST5	2.22±1.09^a^	<2^a^	<2±0.66^c^
	Pre-LS/ST10	2.14±1.16^a^	<2^a^	<2±0.54^c^
VAC	Post-HS	2.76±1.41^a^	<2^a^	2.09±1.15^b^
	Pre-LS	2.62±1.39^a^	<2^a^	2.39±1.31^a^
	Pre-LS/ST5	2.52±1.32^a^	<2^a^	2.36±1.32^a^
	Pre-LS/ST10	2.61±1.31^a^	<2^a^	2.17±1.06^ab^
Storage day (wk)				
0		<2±0.04^d^	<2^a^	<2±0.08^c^
2		2.29±0.62^c^	<2^a^	<2±0.32^b^
4		2.81±0.96^b^	<2^a^	2.13±0.68^b^
8		3.34±1.11^a^	<2^a^	2.78±1.23^a^

1) Treatment: Post-HS = post-rigor-manufactured pork loin ham containing 1.5% salt; Pre-LS = pre-rigor-manufactured pork loin ham containing 0.8% salt; Pre-LS/ST5 = pre-rigor-manufactured pork loin ham containing 0.8% salt and 5% sea tangle extract; Pre-LS/ST10 = pre-rigor-manufactured pork loin ham containing 0.8% salt and 10% sea tangle extract.

a–d Mean values with different superscripts within the same column indicate significant differences (p<0.05).

TPC, total plate count; VRB, *Enterobacteriaceae*; LAB, lactic acid bacteria; MAP, modified atmosphere packaging; VAC, vacuum packaging.

## References

[1] Keast RSJ, Breslin PAS (2002). Modifying the bitterness of selected oral pharmaceuticals with cation and anion series of salts. Pharm Res.

[2] Nissen H, Sørheim O, Dainty R (1996). Effects of vacuum, modified atmospheres and storage temperature on the microbial flora of packaged beef. Food Microbiol.

[3] Lorenzo JM, Sineiro J, Amado IR, Franco D (2014). Influence of natural extracts on the shelf life of modified atmosphere-packaged pork patties. Meat Sci.

[4] Metaxopoulos J, Mataragas M, Drosinos EH (2002). Microbial interaction in cooked cured meat products under vacuum or modified atmosphere at 4°C. J Appl Microbiol.

[5] Kameník J, Saláková A, Pavlík Z, Bořilová G, Hulanková R, Steinhauserová I (2014). Vacuum skin packaging and its effect on selected properties of beef and pork meat. Eur Food Res Technol.

[6] Laury A, Sebranek JG (2007). Use of carbon monoxide combined with carbon dioxide for modified atmosphere packaging of pre- and postrigor fresh pork sausage to improve shelf life. J Food Prot.

[7] Barbieri G, Barbieri G, Bergamaschi M, Francheschini M, Berizi E (2016). Reduction of NaCl in cooked ham by modification of the cooking process and addition of seaweed extract (Palmaria palmata). LWT-Food Sci Technol.

[8] Moroney NC, O’Grady MN, O’Doherty JV, Kerry JP (2012). Addition of seaweed (Laminaria digitata) extracts containing laminarin and fucoidan to porcine diets: influence on the quality and shelf-life of fresh pork. Meat Sci.

[9] Kim HW, Choi JH, Choi YS (2010). Effects of sea tangle (Lamina japonica) powder on quality characteristics of breakfast sausages. Food Sci Anim Resour.

[10] Claus JR, Sørheim O (2006). Preserving pre-rigor meat functionality for beef patty production. Meat Sci.

[11] Aaslyng MD, Bejerholm C, Ertbjerg P, Bertram HC, Andersen HJ (2003). Cooking loss and juiciness of pork in relation to raw meat quality and cooking procedure. Food Qual Prefer.

[12] Juncher D, Rønn B, Mortensen E (2001). Effect of pre-slaughter physiological conditions on the oxidative stability of colour and lipid during chill storage of pork. Meat Sci.

[13] Zhao F, Wei Z, Zhou G, Kristiansen K, Wang C (2022). Effects of different storage temperatures on bacterial communities and functional potential in pork meat. Foods.

[14] Jauregui CA, Regenstein JM, Baker RC (1981). A simple centrifugal method for measuring expressible moisture, a water-binding property of muscle foods. J Food Sci.

[15] Sinnhuber RO, Yu TC (1977). The 2-thiobarbituric acid reaction, an objective measure of the oxidative deterioration occurring in fats and oils. J Jpn Oil Chem Soc.

[16] Conway EJ, Conway EJ (1962). Determination of volatile amines. Microdiffusion analysis and volumetric error.

[17] Ham YK, Song DH, Ha JH (2020). Efficacy of ascorbic acid on processing characteristics and lipid oxidation of pre-rigor salted chicken breasts during vacuum refrigerated storage. LWT-Food Sci Technol.

[18] Keenan DF, Desmond EM, Hayes JE, Kenny TA, Kerry JP (2010). The effect of hot-boning and reduced added phosphate on the processing and sensory properties of cured beef prepared from two forequarter muscles. Meat Sci.

[19] Rajauria G, Jaiswal AK, Abu-Ghannam N, Gupta S (2010). Effect of hydrothermal processing on colour, antioxidant and free radical scavenging capacities of edible Irish brown seaweeds. Int J Food Sci Technol.

[20] Iulietto MF, Sechi P, Borgogni E, Cenci-Goga BT (2015). Meat spoilage: a critical review of a neglected alteration due to ropy slime producing bacteria. Ital J Anim Sci.

[21] Thomas R, Anjaneyulu ASR, Kondaiah N (2008). Effect of hot-boned pork on the quality of hurdle treated pork sausages during ambient temperature (37 ± 1 °C) storage. Food Chem.

[22] Torres E, Pearson AM, Gray JI, Booren AM, Shimokomaki M (1988). Effect of salt on oxidative changes in pre- and post-rigor ground beef. Meat Sci.

[23] Pérez-Alvarez JA, Sanchez-RodrÍguez E, Fernandez-Lopez J (1997). Chemical and color characteristics of “Lomo embuchado” during salting seasoning. J Muscle Foods.

[24] Fernández-López J, Pérez-Alvarez JA, Sayas-Barberá E, Aranda-Catalá V (2000). Characterization of the different states of myoglobin in pork using color parameters and reflectance ratios. J Muscle Foods.

[25] Sørheim O, Kropf DH, Hunt MC, Karwoski MT, Warren KE (1996). Effects of modified gas atmosphere packaging on pork loin colour, display life and drip loss. Meat Sci.

[26] Krause TR, Sebranek JG, Rust RE, Honeyman MS (2006). Use of carbon monoxide packaging for improving the shelf life of pork. J Food Sci.

[27] Zhang M, Sundar S (2005). Effect of oxygen concentration on the shelf-life of fresh pork packed in a modified atmosphere. Packag Technol Sci.

[28] Georgantelis D, Ambrosiadis I, Katikou P, Blekas G, Georgakis SA (2007). Effect of rosemary extract, chitosan and α-tocopherol on microbiological parameters and lipid oxidation of fresh pork sausages stored at 4°C. Meat Sci.

[29] Zhu Y, Li F, Tang J, Wang TT, Jiao Y (2019). Effects of radio frequency, air and water tempering, and different end-point tempering temperatures on pork quality. J Food Process Eng.

[30] Duranton F, Simonin H, Chéret R, Guillou S, de Lamballerie M (2012). Effect of high pressure and salt on pork meat quality and microstructure. J Food Sci.

[31] Desmond E (2006). Reducing salt: a challenge for the meat industry. Meat Sci.

[32] Aberle ED, Forrest JC, Gerrard DE, Mills EW (2001). Principles of meat science.

[33] Schäfer A, Rosenvold K, Purslow PP, Andersen HJ, Henckel P (2002). Physiological and structural events post mortem of importance for drip loss in pork. Meat Sci.

[34] Lee JK, Choi HS, Yoon SK, Kim WJ (1993). Effect of extraction temperature on some quality of sea tangle extract. J Korean Soc Food Sci Nutr.

[35] King AH, Glucksman M (1982). Brown seaweed extracts (Alginates). Food hydrocolloids.

[36] Bernal VM, Smajda CH, Smith JL, Stanley DW (1987). Interactions in protein/polysaccharide/calcium gels. J Food Sci.

[37] Zhang B, Liu Y, Wang H, Liu W, Cheong K, Teng B (2021). Effect of sodium alginate-agar coating containing ginger essential oil on the shelf life and quality of beef. Food Control.

